# Supplementary material for: Multiple variants of the type VII secretion system in Gram-positive bacteria
Source: Microlife. 2024 Jun 5;5:uqae013. doi: 10.1093/femsml/uqae013 (PMC11217815; doi:10.1093/femsml/uqae013)
Supplement: uqae013_Supplemental_Files [file uqae013_supplemental_files.zip › GarrettSuppFigs.pdf]

```
S. aureus     MHKLIIKYNKQLKMLNLRD---GKTYTISEDERADITLKSL---GEVIHLEQNNQG----   50
B. subtilis   MSLLWVFYQNNVQKLNLSNLPSSHPVTIGPDVKDSVTISTIPFNSGVISLKRKESGGQYE   60
B. anthracis  ------------------------------------------------------------   0

S. aureus     TWQANHT----SINKVLVRKGDLDDITLQLYTEADYASFAYPSIQDTMTIGPNAYD-DMV   105
B. subtilis   VFLGNDCLGTIETDISFTLQTDQQDIRLILTGSEPEKSVYFTGNRDEIVCSSEKTNADIY   120
B. anthracis  ------------------------------------------------------------   0

S. aureus     IQSLMNAIIIKDFQSIQESQYVRI-------VHDKNTDVYINYELQEQLTNKAYI--GDH   156
B. subtilis   LNP-------QDFAFAEQSTFSLLRAGGSWSVRPESGTIFLN---GEKINANTPLKPGDE   170
B. anthracis  ------------------------------------------------------------   0

S. aureus     IYVEGIWLEVQADGLNVLSQNTVASSLIRLTQEMPHAQADDYNTYHRSPRIIHREPTDDI   216
B. subtilis   IFWNFTQMRVTEQDLLEIVHYAQFETALTETVKPSTEMQKKYPQYRRTPRMVYDLPDDRV   230
B. anthracis  --------------------------------------MQKSPYFQRSPRVKVDIPTGDV   22

S. aureus     KIERPPQPIQKNNTVIWRSIIPPLVMIALTVVIFL----VRPIGIYILMMIGMSTVTI--   270
B. subtilis   SFSFPSQESDQTNRGLWLVILPPLVMLIVMGVVAI----IQPRGIFILVSLAMFMMTL--   284
B. anthracis  IIHDPPNIPEEPKFSIETMLLPAMMTVLTLVLYFVMMKFMKMNSSYMPLMMVSSIPMLGS   82

S. aureus     -VFGITTYFSEKKKYNKDVEKREKDYKAYLDNKSKEINKAIKAQRFSLNYHYPTVAEIKD   329
B. subtilis   -ITSTVQYFRDKNQRKKREEKRERVYKLYLDNKRKELQALAEKQKQVLEFHFPSFEQMKY   343
B. anthracis  YVITTMGHFRKKKEHRQMVEQLQTRYLEQIQKHRVELDTLKVEQAKYLITQNPSPLKSVQ   141

S. aureus     IVETKAPRIYEKTSHHHDFLHYKLGIANVEKSFKLDYQEEEFNQRR-DELFDDAKELYEF   388
B. subtilis   LTSEISDRIWEKSLESKDYLQLRLGTGTVPSSYEINMSGGDLANRDIDDLMEKSQHMQRV   403
B. anthracis  RIENRESNLWERTPESPDFLDIRIGTG--ERPFLVELKVPEQKGYEENPLVTEAQNVKRD   200

S. aureus     YTDVEQAPLINDLNHGP-IAYIGARHLILEELEKMLIQLSTFHSYHDLEFLFVTREDEVE   447
B. subtilis   YKDIRNAPVTVDLAEGP-MGLVGKSQIVKNEIHQLIGQLSFFNSYHDLRFVFIFHEEEYK   462
B. anthracis  FNTIPNGHISISLKKNDVIGVVGNKEDRLNFIRIVTTQIMTHHAPNEVKIAAFYHEKEKK   260

S. aureus     TLKWARWLPHMTLRGQNIRGFVYNQRTRDQILTSIYSMIKERIQAVRERSRSNEQIIFTP   507
B. subtilis   DWEWMKWLPQFQMPHIYAKGFIYNEQTRDQLLSSLYELIRER-----DLEDDKEKLQFKP   517
B. anthracis  QWDWMRWLPHVWDEQRSMRFLSENQQDAQKLAEVLFTPLNMR-RIYNSSAQADAKVPLIP   319

S. aureus     QLVFVITDMSLIIDHVILEYVNQDLSEYGISLIFVEDVIESLPEHVDTIIDIKSRTEGEL   567
B. subtilis   HFVFVITNQQLISEHVILEYLEGQHEHLGISTIVAAETKESLSENITTLVRYINEHEGDI   577
B. anthracis  MYVFFLSAREFLEDDPLTPMLLREGESVGASTFIFAEQKERLPMECDLVISLNGEN-GEL   378

S. aureus     I------TKEKELVQLKFTPENIDNVDKEYIARRLANLIHVEHLKNAIPDSITFLEMYNV   621
B. subtilis   L------IQKKKAVRIPFRLDHHQREDNERFSRTLRTLNHQVGITNSIPETVSFLELFHA   631
B. anthracis  VETFSSSAENSGTTRASFKVDRLSFERCELGARSIAPIRMKSSTAANIPKVLTFLDLFQA   438

S. aureus     KEVDQLDVVNRWRQNETYKTMAVPLGVRGKDDILSLNLHEKA----HGPHGLVAGTTGSG   677
B. subtilis   KEVKEIGIQQRWLTSESSKSLSVPIGYKGKDDIVYLNLHEKA----HGPHGLLAGTTGSG   687
B. anthracis  KNMEELQVLERWKENRYPTSLPVPIGVREGSKPVFLNIHDKIEKKGHGPHGLMAGTTGSG   498

S. aureus     KSEIIQSYILSLAINFHPHEVAFLLIDYKGGGMANLFKDLVHLVGTITNLDGDE--AMRA   735
B. subtilis   KSEFLQTYILSLAVHFHPHEAAFLLIDYKGGGMAQPFRNIPHLLGTITNIEGSKNFSMRA   747
B. anthracis  KSEVIQSIIAALAATYHPHEMAFMLIDYKGGGMSNTFAGLPHIIASITNLE-DPNLIERA   557

S. aureus     LTSIKAELRKRQRLFGEH-DVNHINQYHKLFKEGIATEPMPHLFIISDEFAELKSEQPDF   794
B. subtilis   LASIKSELKKRQRLFDQY-QVNHINDYTKLYKQGKAEVAMPHLFLISDEFAELKSEEPDF   806
B. anthracis  RISLKAELERRQKLFIQAGNVQHLDEYYE--TSWREKEPLPHLFIVIDEFAQMKKEQPEF   615

S. aureus     MKELVSTARIGRSLGIHLILATQKPSGVVDDQIWSNSKFKLALKVQDRQDSNEILKTPDA   854
B. subtilis   IRELVSAARIGRSLGVHLILATQKPGGIIDDQIWSNSRFKVALKVQDATDSKEILKNSDA   866
B. anthracis  MDELISVAAIGRTLGVHLLLATQKPSGVVNDKIWSNSRFRICLRVQDDADSREMLKIPDA   675

S. aureus     ADITLPGRAYLQVGNNEIYELFQSAWSGATYDIEGDKLEVEDKTIYMINDYGQLQAINKD   914
B. subtilis   ANITVTGRGYLQVGNNEVYELFQSAWSGAPYL---EEVYGTEDEIAIVTDTGLIPLSEVD   923
B. anthracis  SKINVPGRGYLQVGSNEVLELFQSAWSGAPYN-------PDEEKVLDIVDFTEVKLSGER   728
```

```
S. aureus      LSGLE-DEETKENQTELEAVIDHIESITTRLEIEEVKRPWLPPLPENVYQEDLVETDFRK   973
B. subtilis    TE----DNAKKDVQTEIEAVVDEIERIQDEMGIEKLPSPWLPPLAERIPRTLF-------   972
B. anthracis   IKVKKRPKPMTNSPKQLQAFIQYVQSVSEKENIKALPGPWLDPLPEKLLLKEFYAMENWT   788

S. aureus      L--WSDDAKEVELTLGLKDVPEEQYQGPMVLQLKKAGHIALIGSPGYGRTTFLHNIIFDV   1031
B. subtilis    ---PSNEKDHFHFA--YVDEPDLQRQAPIAYKMMEDGNIGIFGSSGYGKSIAAATFLMSF   1027
B. anthracis   IADWNKPKEYLQVTVGLIDDVANQAQYPLKLDLQE-GHLNIYGMPGTGKTTMLQTIIMSL   847

S. aureus      ARHHRPDQAHMYLFDFGTNGLMPVTDIPHVADYFTVDQEDKIAKAIRIFNDEIDRRKKIL   1091
B. subtilis    ADVYTPEELHVYIFDFGNGTLLPLAKLPHTADYFLMDQSRKIEKFMIRIKEEIDRRKRLF   1087
B. anthracis   AVSHTPEEVNFYVIDFGR-MFLDFRDLPHIGGIIQEGENEKMKRLFGFLKQEITNRKESF   906

S. aureus      SQYRVTSISEYRKLTGETIPHVFILIDNFDAVKDSPFQEVFENMMIK-MTREGLALDMQV   1150
B. subtilis    REKEISHIKMYNALSEEELPFIFITIDNFDIVKDEMHE--LESEFVQ-LSRDGQSLGIYF   1144
B. anthracis   SNIGAKSFSMYNRMVEKKVPAIVVMVDGYMRFKNEFEK---ENEVLELLLRESSTYGVYF   963

S. aureus      TLTASRANAMKTPMYINMKTRIAMFLYDKSEVSNVVGQQKFAVKDVV-GRALLSSDDNVS   1209
B. subtilis    MLTATRVNAVRQSLLNNLKTKIVHYLMDQSEGYSIYGRPKFNLEPIP-GRVIIQKEELYF   1203
B. anthracis   YFSLNQTIDMFDRVRNNIPMALTFELQDGTEYHNLVGRPKFPLIEVPVGRGLMKGQPPEL   1023

S. aureus      FHIGQPFKHDETKSYNDQINDEVSAMT-EFYKGETPNDIPMMPDEIKYEDYRESLNLPDI   1268
B. subtilis    AQMFLPVDADDDIGMFNELKSDVQKLQGRFASMEQPAPIPMLPESLSTRELSIRFKLERK   1263
B. anthracis   FQAALPFIGESELEYSQQLKTIIQKMNGEW-NGEKAKTIPMVPKEIFVEHMVGKLETAQI   1082

S. aureus      VANGALPIGLDYEGVTLQKIKLTE--PAMISSENPREIAHIAEIMMKEID----------   1316
B. subtilis    PL--SVPIGLHEETVSPVYFDLGKHKHCLILGQTQRGKTNVLKVMLEHLI-DDETEMIGL   1320
B. anthracis   SA------GIETEDIRLQSFSLDEMSHIFIGGRIEGGKTSLLQTLLFTTTYQYSPEKVEL   1136

S. aureus      --ILNEKYAICIADSSGEFKAYRHQVANFAEERE------DIKAIHQLMI-EDLKQRE-M   1366
B. subtilis    FDSIDRGLSHYAKESDVSYLETKEDIEQWIETAE------DIFKTREAMYVEAVRQGDAQ   1374
B. anthracis   Y-LVDLGERPTGILALGDLPHVKKKVTDAIQLKEMLDELLELINNREAVM-PTLDPNVTV   1194

S. aureus      DGPFEKDSLYI--INDFKTFIDCTYIPEDDVKKLITKGPELGLNILFVGIHKELID-AYD   1423
B. subtilis    NLRFSQVVLMIDGITRFQQTIDTRI--QDRLANFMKSYAHLGFSFIPGGNHSEFSK-GYD   1431
B. anthracis   EFPYKRIMIAIDDIDQMLTVLSTDYEAKNKMELIVQNCKNKGVHFMTAATTSSLNSYSHE   1254

S. aureus      KQIDVARKMINQFSIGIRISDQQFF---KFRFIQREPVIKENEAYMVANQ------AYQK   1474
B. subtilis    SLTTEMKQIRHAILL-MKKSEQNVI---PLPYQRQEPEIQPGFGYVVENG------KEQK   1481
B. anthracis   KWFAEIRKRSIGYLLGTTQNNDVYFFNMKLPHTEMDQELLSGDGYCIRRKPIKIKCAYTP   1314

S. aureus      IRWFK-----------------                                       1479
B. subtilis    VQIPLCSAERESAR--------                                       1495
B. anthracis   LHLLRTMIDKISVKWTELKVK                                        1335
```

Figure S1. Sequence alignment of the EssC sequences from *B. subtilis* and *S. aureus* with the protein annotated as EssC from *B. anthracis*. The region boxed in green is the FHA domains, in purple is the two transmembrane domains and in yellow the four NTPase domains.

```
EccC/1-1391 ------------------------------------------------------------
EssC/1-1479 -------MHKLIIKYNKQLKMLNLRDGKT--------------YTISEDERADITLKSLG
TscC/1-1328 ------------------------------------------------------------
TsdC/1-1563 ---MSVDAYTVTMIDAQRLENFALSQ-ES-----LGVSHLAFSESGPGYPCADVHIDGEH
TseC/1-1572 --------MNVTLINKERIHSISLPEKVR--------GQYWIYDSNGKSSRKLIGIEGVN
TsfC/1-1544 MEEKRNYNNVLILYNSDFYKEIDLDDYEKGTVVVGNSANCDIKLKLETEELVYIYFNKIN
TsgC/1-1293 ------------------------------------------------------------
TshC/1-1330 ------------------------------------------------------------
TsiC/1-1522 -----MKEMILYVYYNDTVKEYILSNESS---------------ICNIELSSLLPVVIVN
TsjC/1-1525 ------MNMKLLVYSDKKLYEVKLGNSKT-AVTIGNRKRFKHYIETEEKNRIDMSIKPIK

EccC/1-1391 ------------------------------------------------------------
EssC/1-1479 EVIHLEQNNQGTWQANHTSINKVL--------------------VRKGDLDDITLQLYTE
TscC/1-1328 ------------------------------------------------------------
TsdC/1-1563 MRLCAGCGSRLFDESGIIDSYDMHGVSEGHVLTLRTESTHSSGQSGNPGVASLLFIRPST
TseC/1-1572 GEWILKSNKDVKVLDNTGKPIRNTVL---------QPLSIYNLGLSTQDQKTIIFTEPTT
TsfC/1-1544 TSWQIKDGENAYCIVNDIKTPRKILVNG-------DQITIKNVKDKEELFKINFFIDFIV
TsgC/1-1293 ------------------------------------------------------------
TshC/1-1330 ------------------------------------------------------------
TsiC/1-1522 GEWFLAPTEDMAVSKKQINVGDKI-----------------SVSITDTDEKATIYVDRET
TsjC/1-1525 DIWVLKSKYYIGAEGQEVDIKLEHGV---------------VLQFGLNGKSSIMAIAFSE

EccC/1-1391 ------------------------------------------------------------
EssC/1-1479 ADYASFAYPSIQDT--MTIGPNAYDDMVIQSLMNAIIIKDFQSIQESQYVRIVHDKNTDV
TscC/1-1328 ------------------------------------------------------------
TsdC/1-1563 AGAREFRKIAFSSDADMTIGRDGSSALVYSLPFVSEHHAVLSYRDAE-FSIVDNGSVNGT
TseC/1-1572 DDRQIFTKYLVDRDIEITIGRTEQNDIVLANRFVSASHAKLSFHSGQ-WRIEDQNSSNGT
TsfC/1-1544 EKENYDKVISLKDVTYLSIGRDEDNNISIRDDLIDRKHCEIKCDSNNKFYVTDLKSKYGV
TsgC/1-1293 ------------------------------------------------------------
TshC/1-1330 ------------------------------------------------------------
TsiC/1-1522 NIELTYKKYSIEGEKQILIGNSEKNTIVYNGFDMKPNHAVISIENGR--ATISTKGNKCL
TsjC/1-1525 DKLLSKEKLMLKEKVKFTIGRGKFNDIVFNDIKVSEKHAEIFEDDGK-YVLVDLNSTNKT
                                   GR                SXXH              NG

EccC/1-1391 ------------------------------------------------------------
EssC/1-1479 YINYELQEQLT-NKAYIGDHIYVEGIWLEVQADGLNVLSQNTVA-------------SSL
TscC/1-1328 ------------------------------------------------------------
TsdC/1-1563 FINGVRLTSGRPVHLNAGDLVQIMDLIFTVGLRFISLNNPQGVRLAQSPAWHMVSHESIL
TseC/1-1572 FVDGERVKS---KALKIGDTIYIMGYKMIIGSYFVAFNNPDGTLTIKTHALKPFIPQTV-
TsfC/1-1544 YIDGKKVEDK--VYLKDNNFITICGHKILFMDNSLALSNLNENIVINGA-----------
TsgC/1-1293 ------------------------------------------------------------
TshC/1-1330 ------------------------------------------------------------
TsiC/1-1522 CVNGKMTES---TVLSYGDFIHIMGLKMFYLGKSLAINDSIHLGQCNLSPYTPIKPRE--
TsjC/1-1525 YLNGEMITR---AILARGDEINICGYIIQFNDESIVVQGALKN-----------------

EccC/1-1391 ------------MKRGFARPTPEKPPVIKPENIVLSTPLSIPPPEGKPWWLIVVGVVVVG
EssC/1-1479 IRLTQEMPHAQADDYNTYHRSPRIIHREPTDDIKIERPPQPIQKNNTVIWRSIIPPLVM-
TscC/1-1328 -------------MNVLYQRSPRIKPTLKEDSIEIPKPQNEPSKPSFSLISILLPAVMTI
TsdC/1-1563 AASPPAAQADDVEEAKPFYPAPRLMHTVHVKAFHVDEPPQAMKREEQPALMQLGPSFLMG
TseC/1-1572 -ETPDEEEEYEVPEVDYFYRSPRFKRDVEKAVIKIDSPPPNAIGEEMPMMLVIGPSMTMG
TsfC/1-1544 -TVYNKDRFDKYMSYPDFYRSPRFLVELPRDEIEIDAPPDKPSKVSIQYLLSVIPIVGT-
TsgC/1-1293 ----------MINEKYEFSRQPRFLPEMPRGELEIPNPPSAYDKPEIAWFALLAPPAVM-
TshC/1-1330 -------MDIKSASDTQIQRTPRIMPYYPDGELVLQDPARPLGKPTFSWLTILVPPLAM-
TsiC/1-1522 -DIKGKKKKKTKKEDLYFQRSPRIIKKLEEGMMDIDSPPSPPEQKEQPLILTIGPALTMG
TsjC/1-1525 -TKQKAGQEKVVTEYPFVQRAPRIYPEVKREDLKIENPPTLPNKPSIYGLMTVLPSLIF-

EccC/1-1391 LLGGMVAMVFASGSHVFGGIGSIFPLFMMVGIM---MMMFRGMGGGQQQMSRPKLDAMRA
EssC/1-1479 --IALTVVI----FLVRPIGIYILMMIGMSTV----TIVFGITTYFSEKKKYNKDVEKRE
TscC/1-1328 FSIGFYIYMNL--TGKTGNSSYMMFQMVSVMMMLT-SYTIPFFVYLGNKKKYKEQLKQRV
TsdC/1-1563 IGSVFMAISAVSNLSRGGNVLSAAPSLAMAVAMVGGTLIWPMISKRYTKKVEETKERRRQ
TseC/1-1572 MASMATGLFAVNHAMSTGDISKAIPSIVMSCSMLLGTVLWPILTKKFDMRRRRKKEKIRQ
TsfC/1-1544 --VVMMSLMS---SSMGSNKTYMIYSVGMVSL----SALVSVITFVTEFVSSKKYKKKRN
TsgC/1-1293 --LVISMLL-----AMTSNSRYMLLSISMTIM----TLIVSLTGAASQIKKYKKKKKERE
TshC/1-1330 --LVVAIFM----STLTKSHIMLISTIGMTTV----TVVVSILNYRSTVKKHEEQTKKQE
TsiC/1-1522 MAMMVSMMFTV--YSSRNNPFMVIPGIAMTGSMLLGAVLWPILSRRHKKKSKRKEEKKRI
TsjC/1-1525 --FVVTIIT----SMKSNNGSSTIIFAAGTGA----SALVYGGSYIGQKIVYNKKVKKRN
```

```
EccC/1-1391 Q-FMLMLDMLRETAQESADSMDANYRWFHPAPNTLAAAVG--SPRMWERKPDGKDLNFGV
EssC/1-1479 KDYKAYLDNKSKEINKAIKAQRFSLNYHYPTVAEIKDIVETKAPRIYEKTSHHHD--FLH
TscC/1-1328 RLYHEELDKYREQLIAGHKEQVDVLFEVHGDPDVCFQVVKNRSSVLWERSAHDDD--FLH
TsdC/1-1563 DKYTDYLNSITAKLLEERDRQSSILNENRPPLQNVLQRAYDLSPHLMDRNATQAD--FLQ
TseC/1-1572 EKYKDYLDRIAVVFNEEAEKQEEILRENHVPISDCINRIENVQRNLWERGPGQND--FLK
TsfC/1-1544 KFYRGYIAEKEKEITDNIELQKKNLKIMNPEINECFDFVREFDRRLWEKKPSHPD--FLN
TsgC/1-1293 KKYLQFIADSRSELQLAREQQIKAMNEMNPDPNACLQRIQELDNRLWERTPSSPD--FLS
TshC/1-1330 KKYLDYLFNIRYELLEASNQQREALRVISPSIDECISIVKQRQKQLWERTAIETD--FLS
TsiC/1-1522 KRYRKYMQNEYSRLEEKIAYNKKVLLGTYPEPTVLISRALNRDRRLWERMPSNKD--FLD
TsjC/1-1525 SKYTIYTNEIEKKLLKAEKNLREVLFDENPDLSEAIEIVKNLNMKLWSRSYGDED--FLI

EccC/1-1391 VRVGVGMTRPEVTWGEPQNMPTDIELEPVTGKALQEFGRYQSVVYNLPKMVSLLVEPWYA
EssC/1-1479 YKLGIANVEKSFKLDYQEE-EFNQRRDELFDDAKE-LYEFYTDVEQAPLINDL-NHGPIA
TscC/1-1328 TRIGTGNLPFYIEVKAPR--PEGYVKDSLIEAAQE-LAGEFQTVADAPVALPLFQAKVIG
TsdC/1-1563 LRVGMGEQRVEAQVQAPQR-RFSMENDALLEQAEA-LADKPTMMQ-APVAVDLAGPGTLG
TseC/1-1572 LRVGTGSGLLAADLTYSEK-KFSIDDDNLQGELYT-LVESPKVLKNIPITLSLYENHISG
TsfC/1-1544 VMLGHGDTSISFSVKIPDN-KNKMEIDDLERLAPE-LKKKYQKVEDVPLSLNLLKSSPIG
TsgC/1-1293 LRLGVGSVPAALRINYTKQ-AIIMETDPLLMEPQR-LALEFDRVQGVPVSVDLIASEICG
TshC/1-1330 VRMGNGMQPLALNPTYTSN-TKAVDEDNPLEKIASTICEEMAFVQDVPVQIPLQSISTLG
TsiC/1-1522 IRLGTGTVPSMINIQKPKD-RFTMRDDNLMGELKD-IGNNFDGVSGMPIAFSMMGNNMLG
TsjC/1-1525 LRVGLGNEEFPLKI-IKEK-RNFFDEDTLLDRLDK-ITEKYNIVDNVPICIPIKSNGITA

EccC/1-1391 LVGEREQVLGLMRAIICQLAFSHGPDHVQMIVVSS--DLDQWDWVKWLPH-FGDSRRHDA
EssC/1-1479 YIGARHLILEELEKMLIQLSTFHSYHDLEFLFVTREDEVETLKWARWLPHMTLRGQNIRG
TscC/1-1328 IVGDKEAVMNSLRVMIAQLAVRHSPDEVKLAAFYEEKDAEDWDWMRWLPHTWDEHRSQRY
TsdC/1-1563 VVGARPVVWDFVRGLLAQTCSFYGYNEVKIVAIVDPADQAQWAFLKDVPHARSDDGKMRY
TseC/1-1572 VIGTRKQTIEFAKGLIFQLSALYSYDEVKMIFIYDQDEEAQFSFAKWLPHVWSNDNKFRF
TsfC/1-1544 VVGKKQHTYKFISNLMVKLTAGHFYEDVKIAIIVSNEDKESLSWVRWVPHIWSRKKEIRF
TsgC/1-1293 IAGEEDKTFDMVTQMLLQLITHHGYDDVRVVILASEEGLEKWDWLKHLPHLWSEGYGIRF
TshC/1-1330 LFGKRAEVSEFLNALLVHLTTHHGFDDVKIVGLFDQAEMEMWGWTKWLPHTWDKDREIRF
TsiC/1-1522 MIGNRKTTIDTAMASIVHISALHSYDEVKIVCVYNAEESHKWEWVKQLPHVWAPEKALRF
TsjC/1-1525 VIGNRDKVVETVKNMLLQISINHTPDEVKLVVISDEKENKHWDWAKWLPHVWDDDKNMRF

EccC/1-1391 AGNARMVYTSVREFAAEQAELFAGR-GSFTPRHASSSAQTPTPHTVIIAD-----VDDPQ
EssC/1-1479 FVYNQ---RTRDQILTSIYSMIKERIQAVRERSRSNEQIIFTPQLVFVITDMSLIIDHVI
TscC/1-1328 MSDRR---SSAHQLADELLQRLSRR-KTARHE-QRKKTVEL-PVQVVLLSSGQLIEEEPL
TsdC/1-1563 LASDQ---AELLGLDSMLSKALAER-DGVRGD-AGKACI---PFYVVVCANRELAAASSS
TseC/1-1572 VATND---NEVKEVSAYIEKEIEIR-ANTNES--EMEDVK--PYYIVFALSKELAFKAEM
TsfC/1-1544 IGSDK---ESSHNVLNYLYDVARER-SDDNEN-SHEPKVNL-PHFIVFIADNKLTENEPI
TsgC/1-1293 LLCGK---AIAHAVFGELNTVFKER------EM-KKLGGIPL-PHYVFIIEESSLLEEEPI
TshC/1-1330 LASNE---YEASILADVLLAEMKRR-DEDKPS-YGTKTIKL-PHYVFLMLAPTLWEDSDL
TsiC/1-1522 VASSR---DEVRDVFLYLKEVLSDR-DETKDNGYDKQVMHL-PHFLVYIADPELVEDELV
TsjC/1-1525 MAKNK---EQAHKLLSNLYDVLSER-KNKLEDKNNYDSYRFSPHFVFVLASRELIQNEAI

EccC/1-1391 WEYVISAEGVDGVTFFDLTGSSMWTDIPERKLQFDKTGVIEALPRDR-------------
EssC/1-1479 LEYVNQDLSEYGISLI------FVEDVIE-SLPEHVDTIIDIKSRTE-------------
TscC/1-1328 LPLLLEDAAEIDACTI------ILSERKE-TLPMQCHLIVDVGKE-K-------------
TsdC/1-1563 VSKLGNLEENKGFCVL------FLGDDLR-DLPRECSQVIELAPPDSSQAAKADYALGTR
TseC/1-1572 LKQVYAKKENLHISIV------TFYDELK-NLPKECSMVVELEEN-S-------------
TsfC/1-1544 MPYLENRN-NVNMTAV------FVSESIE-MLPKECTDVIEVSAGYN-------------
TsgC/1-1293 NKYLYNKSASLGISSV------YIARNQA-YLPMNCKQVILV----Q-------------
TshC/1-1330 MKYIVSNNDSLGITSI------FISERIDVALPLNSQVIIEVKNG-Q-------------
TsiC/1-1522 MKYLTNPNKQLGVSTI------FIYDKLN-QLPKECKAFIQCEET-E-------------
TsjC/1-1525 LKYLLNVDADMEISTI------FLSDKLG-NLPRNCNNIVQLNEG-E-------------

EccC/1-1391 ----DTWMVIDDK-----AWFFALTDQVSIAEAEEFAQKLAQWRLAEAYEEIGQRVAHIG
EssC/1-1479 ----GELITKEKELV---QLKFTPEN-IDNVDKEYIARRLANL-IHVEHLKNAIP----D
TscC/1-1328 ----GHYSLKREDGTFE-QMSFVPDN-LGLDKVDALSRYMAPIRLKRSLAS-DIP----E
TsdC/1-1563 GVLAGPSRMFDRSDVGGSERIFQPDILLSAAGIERFVLALAHARLATGRSEGAMP----E
TseC/1-1572 ----GKLFDK-NDITGK-TTSFTPDIYLNGADPYQLSVKLANVPLDTLANSFNLP----Q
TsfC/1-1544 ----GSSTETINRAH---FMQFAFDD-VNLRECYEYSKRMAPINVKSSFSESSLK----S
TsgC/1-1293 ----GKTGELADRETGE-KTTFNPDT-INLQSLSEAVRKMAPLRIKNSGGNFTLP----T
TshC/1-1330 ----GMTRKDQNNCMGQATYHFLSDS-VSCKRAEHFSRMLAPLTIKETKNNSFIP----P
TsiC/1-1522 ----SSLYNRDKPETG--LIKFNNDS-VIGKDLDSYSRALAGIKIKEIASSASLT----S
TsjC/1-1525 ----GIIYNIQNSSE---KSYVTPDK-MDNKMLMEYSRTMAPLRLEKDSYSNKLP----R
```

```
EccC/1-1391 ARDILSYYGIDDPGNIDFDSLWASRTDTMGRSRLRAPFGNRSDNGELLFLDMKSL-DEGG
EssC/1-1479 SITFLEMYNVKEVDQLDVVNRW---RQNETYKTMAVPLGVRG-KDDILSLNLHEK----A
TscC/1-1328 VLTLFEMLNIKTAADLDVERRW---RNNRYPESLPVPFGVRA-GGKNVNLNIHDKIERQG
TsdC/1-1563 SLGFMEMFQKGNVLQLNIADRW---KEHDSSRSLAAQVGIGA-KGEPVSLDLHEE----A
TseC/1-1572 MFTFLELYGVGKVEHLNALTRW---KDNDPTKSLEAPVGVDT-LGELFKLDLHEK----F
TsfC/1-1544 LITLFDLYNVKRTQDFNVLSNW---GKNKVYETMEVPIGVKA-GDEIEYLNLHEK----Y
TsgC/1-1293 SLTLLDKLQAKKTGEIDLLTRW---NMNKPYQGMSVPIGAKA-GGTLFHLDMHET----G
TshC/1-1330 MVTFLDSFNVKLVEELNITNRW---GENQANRTLAVPLGQAS-AGKTLLFDMHEK----N
TsiC/1-1522 MLTFLEMYKIGRIENLSIKSRW---KNNLSYRTLEAPLGIKA-GGTQFLLNIHEK----Y
TsjC/1-1525 SISLFEELDITNVEEIDFNSIW---SQAEAHKTLSVPVGIRE-NGEKFYLDLHQK----Y

EccC/1-1391 DGPHGVMSGTTGSGKSTLVRTVIESLMLSHPPEELQFVLADLKGGSAVKPFAG----VPH
EssC/1-1479 HGPHGLVAGTTGSGKSEIIQSYILSLAINFHPHEVAFLLIDYKGGGMANLFKD----LVH
TscC/1-1328 HGPHGLIAGTTGSGKSEVIQSIVASLAAEFHPHDLAFMLIDYKGGGMSNTFVH----LPH
TsdC/1-1563 HGPHGLIAGTTGSGKSEFIITWVLSMALNYSPDEVAFVLIDYKGGGLAGAFDNARLRLPH
TseC/1-1572 HGPHGLVAGMTGSGKSEFIISYILSLAVNYHPHEVAFILIDYKGGGMAKSFEK----LPH
TsfC/1-1544 HGPHGLVAGTTGSGKSEILQTYIISLAINYHPYDVALIIIDYKGGGMANLFKN----LPH
TsgC/1-1293 HGPHGLVAGTTGSGKSELLQSIIISLAINYHPHDVVFVLIDYKGGGMADVFQG----MPH
TshC/1-1330 YGPHGLVAGTTGSGKSELLQSLLLALAVNYHPHEIAFVLIDYKGGGMANAFAG----LPH
TsiC/1-1522 HGPHGLIAGMTGSGKSEFIQSYILSMAVNYHPHDVAFILIDYKGGGMANCFIG----LPH
TsjC/1-1525 HGPHGLVAGTTGAGKSELLETVIAALSFSYSPEYVNELLIDYKGGSMANVFKN----LPH

EccC/1-1391 VSRIITDLEEDQALMERFLDALWGEIARRKAICDSA----GVDDAK--EYNSVRARMR--
EssC/1-1479 LVGTITNLDGDE--AMRALTSIKAELRKRQRLFGEH----DVNHIN--QYH----KLF--
TscC/1-1328 VVATITNLSSSL--MERAKVSLKAELVRRQKILNDAG---NLQHID--EYY----KLL--
TsdC/1-1563 LAGTITNLDGAA--VARSMVSIKSELKRRQALFNKACEATGEATMDIGKYI----SYF--
TseC/1-1572 TAGIITNLDGAA--IKRSLVSIESELKRRQAIFADASKLVGESNIDIYKYQ----KLY--
TsfC/1-1544 LVGTITNLDGNQ--INRSLVSIKSELKRRQRIFAKC----NVNHID--AYI----KLY--
TsgC/1-1293 LVGTITNLGGNQ--TTRALLSIKSELMRRQRLFSEF----GVNNID--KYQ----KLYYS
TshC/1-1330 LVGTITNLGGNQ--INRALASIKSELLRRQRLFGEA----GVTSID--DYI----VLY--
TsiC/1-1522 ITGTITNLGGNQ--IRRSLVSLQSELKRRQRVFAEH----GVNHID--KYQ----QLY--
TsjC/1-1525 AVGTVTNLEGNG--SKRAIIAIDSEIKRREKILTDN----TYSNID--EYQ----KNY--

EccC/1-1391 -ARGQDMAPLPMLVVVIDEFYEWFRIMPTAVDVLDSIGRQGRAYWIHLMMASQTIESRAE
EssC/1-1479 -KEGIATEPMPHLFIISDEFAELKSEQPDFMKELVSTARIGRSLGIHLILATQKPSGVVD
TscC/1-1328 RREGG--QPLPHLVIIIDEFAQLKKDQPEFMDELISIAAIGRTLGVHLILATQKPAGVVD
TsdC/1-1563 -RQGVLSEPCPHLFVVADEFAELKQQEPTFLDELVSAARIGRSLGVHLVLATQKPTGVVS
TseC/1-1572 -REGVVREPLQHLFIISDEFAELKTQQPEFMTQLVSAARIGRSLGVHLILATQKPSGVVD
TsfC/1-1544 -KEKKVTEPIPHLIIIADEFAELKSDQPEFMAELVSTARIGRSLGVHLILATQKPAGVVD
TsgC/1-1293 RNAGGHMPAIPHLIMIADEFAELKQDQPDFMKELVSAARVGRSLGVHLILATQKPAGVVD
TshC/1-1330 -REHKVQLPLPHLIIVVDEFAELKSDQPEFMKELVSAARVGRSLGIHLILATQKPSGVVD
TsiC/1-1522 -KEGKADCPLPHLVIISDEFAELKAGQPEFMQELVSAARIGRSLGVHLILATQKPSGVVD
TsjC/1-1525 -KFGKHKMSLPHLIIIVDEFAQLKKNDPDFISQLVNVAVVGRSLGVHLILATQKPSGIVD

EccC/1-1391 -KLMENMGYRLVLKARTAGAAQAA-GVPNAVNLPAQAGLGYFRKSLEDIIR-FQAEFLWR
EssC/1-1479 DQIWSNSKFKLALKVQDRQDSNEILKTPDAADITLP-GRAYLQVGNNEIYELFQSAWSGA
TscC/1-1328 DKIWSNSRFRICLRVQDEGDSRDMLKIPNAAWINKP-GRGYFQVGSDELFEEVQFAWSGA
TsdC/1-1563 DQIASNARFRVCLKVADAADSREMIRRTDAAALTRP-GEFYLLVGYDELFTGGQAAYAGG
TseC/1-1572 DQIWSNSKFRVSLKVQERADSMDMLKRPDAAELTDT-GRFYLQVGYNELFEMGQSAWAGA
TsfC/1-1544 NQIWSNSKFKLCLKVQDAEDSKEVLKSSLAADIVEP-GRAYFQVGNNEIFELFQSAWSGA
TsgC/1-1293 DQIWSNSKFKICLKVQDERDSRDVIKRPDAAMIKEP-GRAFIQVGNDEIFELFQSTYSGA
TshC/1-1330 DQIWSNSRFKLCLKVQTPSDSQEMLKRPDAAEIKEK-GRGYLQVGNNEVFTLFQSSWSGA
TsiC/1-1522 DQIWSNTRFRICLKVLDKSDSNEMLKRPEAANIKEP-GRCYVQVGNNEIFELLQSGWSGG
TsjC/1-1525 PQIETNTNLKICLRVQDNEDSRTVIGKPDASSIANP-GRAYIKVGNDLVYELIQTAFAGE

EccC/1-1391 DYFQPGVSIDGEEAPALV--HSIDYIRPQLFTNSFTPLEVSVGGPDIEPVVAQPNGEVLE
EssC/1-1479 TYDIEGDKLEVE--DKTI--YMIND-----------------------------YGQLQA
TscC/1-1328 PYVEQTDS------PGRIVPTEIAL-----------------------------NGKREN
TsdC/1-1563 TYTERDVYEPKR--DVSV--ELVGL-----------------------------DGGAVS
TseC/1-1572 PYYPSDKVVVEK--DTSV--VVIDR-----------------------------NGRPIK
TsfC/1-1544 KKYDEDDVNKN---EVEI--FNVGI-----------------------------DGTRSL
TsgC/1-1293 DYDPDGELQKSENKTKRI--YEVSL-----------------------------NGRIEQ
TshC/1-1330 PYNNTDSEEEV---AIKA--NSLSI-----------------------------NGERKR
TsiC/1-1522 SYIPTDKIENDE--DNQI--SLIDG-----------------------------CGRAIQ
TsjC/1-1525 KYRKTAAKK-----DKKI--LMVDT-----------------------------DGERFD
```

```
EccC/1-1391 SDDIEGGEDEDEEGVRTPKVGTVIIDQLRKIKF-----EPYRL-WQPPLTQPVAIDDLVN
EssC/1-1479 INKD-LSGLEDEETKENQTELEAVIDHIESITTRLEI-EEVKRPWLPPLPENVYQEDLVE
TscC/1-1328 LLTT-SMSAANLQMEQPPKQLQVFIDKLAEAAKEEGI-PRLQGPWLPPLPDRLELEVLP-
TsdC/1-1563 RLRP-PSAGRSGGIS----ELNAVLEQVCEVARAVN--KSARPLWLEPLPERVLLDDLRQ
TseC/1-1572 QAKIDKKKGLFTNPKK---QLDVITDYLSNIAAEENI--KIRPLWLEPIPALILLADIRK
TsfC/1-1544 LYST-KDKEKDKKRIT---QLEAAIEEIDKVCSENHI-ERLDGPWLPPLEDEIYLDDLLK
TsgC/1-1293 IYPRYEEKIVKHELPS---QLGATVEHIVRTAERSNI-EPLKGPWLPPLPELVYIDGILE
TshC/1-1330 LLPK-VSNHNESKRLN---QLESVVKYINRVSEEQNIREAFQL-WLPPLPELLSLQELLV
TsiC/1-1522 TVSC-KPKPVKSSTT----QLTAIVDYISDLAKDEGI-NPLKL-WLDPLEEEVFIQDIED
TsjC/1-1525 IVER-EKEGHSEDVT----QLSKLIDSI-KVYCDLNMNYERKIPWLPPLKSHIYIDKLS-

EccC/1-1391 RFL----GRPWHKEYGS---------------------------------ACNLVFPIGI
EssC/1-1479 TDF----RKLWSDDAKE------------------------------------VELTLGL
TscC/1-1328 -------GIHIHPAAEE------------------------------------LEGLVGI
TsdC/1-1563 KFD-------FRADHAG------------------------------------LTAVVGE
TseC/1-1572 KYN--ADRGTWLSSENRPDVFQEAVSEIAAAAETVQEDVLKSFTTASRRASLKLNPVIGE
TsfC/1-1544 DSKTGFNGKEWIEDREW------------------------------------ICPTIGM
TsgC/1-1293 NKQ---ELGHWAKQENL------------------------------------LKVPAGI
TshC/1-1330 NEE-GWNGQQWTEPGDY------------------------------------LVAPVGL
TsiC/1-1522 RKN-GWNGDDWQEVDHW------------------------------------LCPTIGL
TsjC/1-1525 ----------YIPQDK-------------------------------------FKARIGV

EccC/1-1391 IDRPYKHDQPPWTVDTSGPGANVLILGAGGSGKTTALQTLICSAALTHTPQQVQFYCLAY
EssC/1-1479 KDVPEEQYQGPMVLQLKKAG-HIALIGSPGYGRTTFLHNIIFDVARHHRPDQAHMYLFDF
TscC/1-1328 IDDLPNQSQRPLHIPLQQ-G-HWAVYGMPGLGKTTFVQTLLMSLALRYPPQAWYGYIVDM
TsdC/1-1563 LDDPEHQRQDVFSVDVAEAG-NVALYGTPTSGVESLAMSMVCSLMAEYTPDELNVYVWDM
TseC/1-1572 YDDPVHQQQCLLRLPLTDEG-NTIIYGAAGTGKTTFLNTMVYSLIHEHTPEEVNLYLLDL
TsfC/1-1544 LDDPERQDQRPLKIDLGELG-HLLLVGAPGYGKTTFLQTLMISLMLNYTPEEVNMYILDF
TsgC/1-1293 MDDPRSQSQDLLEIDFANEG-NLFVYGASGTGKTEFLKTLCMSMAHHYSAEDVNIYIMDF
TshC/1-1330 IDNPSQQAQYQLKIDFGRDG-HLLIYGAPSSGKTTMLKTILMSLALKYNPDYVHFYILDF
TsiC/1-1522 IDDPANQNQFPLKLDIGNMG-HVLLVGAPGTGKTTFLQTMIYSLVTSYTPELVNLYLLDF
TsjC/1-1525 YDDPYHQSQEIFEIDIQKEN-HIALYGMAGTGKTTFLQTFILSLASKNSPRDINFYIADC

EccC/1-1391 SSTALTTVSRIPHVGEVAGPTDPYGVRRTVAELLALVRERKRSFLECGIASMEMFRRRKF
EssC/1-1479 GTNGLMPVTDIPHVADYFTVDQEDKIAKAIRIFNDEIDRRKKILSQYRVTSISEYRKLT-
TscC/1-1328 G-RMMRDFAGLPHIGGVMMAEEDDRIKRLFRFLMKTVSVRKEMFAETGVKTAAAYRRSQ-
TsdC/1-1563 GAGSLSALSKAPHVGGVVLVEDSERVDNLFLLLEQEIAHRRRLFSRSG-GSYESYNGLP-
TseC/1-1572 ASETLRAFSKAPHVGDVILSYESEKVSNLFKMLQGEVEKRKKLLADYG-GDHRSYVEAT-
TsfC/1-1544 GARTLKMYEKSAYVGGVVTSDDEEKLMNLIKYLHKEIDRRKKIFSDNGVGSLKAYREVG-
TsgC/1-1293 GGNSLRIFEKLPHVGGVMTIEEKTKIDQFIMFLFREIEERKVLFEQSGSNGFNAYRHS--
TshC/1-1330 GTRTLGVFNDIPHLGDVIYPEDEQKVDKLLQLLLTELEDRKRKFSKLGISNLVAYRSYT-
TsiC/1-1522 GGRTMGYYKYLPHTGGVIFSEESDKLDKLFKMLKKELDNRKKKFSEYGVGTLKAYMEAT-
TsjC/1-1525 DKGTLNMFKSLVHTGEVVLSDDTDKVKKLLKFIMKEIDKRKNALTSMRAISVNDYNYKT-

EccC/1-1391 GGEAGPVPDDGFGDVYLVIDNYRALAE--ENEVLIEQVNVIINQGPSFGVHVVVTADRES
EssC/1-1479 ---GETIP-----HVFILIDNFDAVKDSPFQEVFENMMIKMTREGLALDMQVTLTASRAN
TscC/1-1328 ---AGPLP-----EMLIVIDGYLNFRN--SYPDENEMLEFILREGGNLGITFVITSNRIS
TsdC/1-1563 AEDRDPVP-----RIVVVLADIAAFNE--GYEKYVDRLNTLARDAPRYGIHLIITASLWS
TseC/1-1572 ---GQKVP-----AIVVAIHNFSAFTE--IYDEKEEAVSYLSREGTKYGIYFVLTALGTG
TsfC/1-1544 ---NTLIP-----QIVIILDNYSALIE--FYQDLEDELIFLSREGGTLGISLVVTAGNYT
TsgC/1-1293 ---GRKLT-----SIVLMIDNYFALSE--SYEDLDAHMMLLAREGTKYGIYLVATASNAT
TshC/1-1330 ---KEEIP-----SIVVALDNYTGFAE--SYVEQVLELGKLVREGGNYGIYFVFTGNAIN
TsiC/1-1522 ---NAKIP-----AMIVILDNYEAFYE--LYSDYEGILTTFSREGGNYGIYMVITASNAN
TsjC/1-1525 ---GKVLP-----QIIFIIDDIVTLLT--VNDDFKEDIVKIVREGGALGVHMVYTANSSN

EccC/1-1391 ELRPPVRSGFGSRIELRLAAVEDAKLVRSRFAKDVPVKP-GRGMVAVNYVRLDSDPQAGL
EssC/1-1479 AMKTPMYINMKTRIAMFLYDKSEVSNVVGQQKFAVKDVV-GRALLSSD-DNVSFH-----
TscC/1-1328 DIFEKVRSNIASAVTFELADPSDYYYAVGRPARNPGSMPPGRGLVKGNVPPLEFQ-----
TsdC/1-1563 QVNMRLRASMGRTLVTSFSNNDDYNSVLNGMHGVTPPKGFLAGVFQNGKGVTVYQ-----
TseC/1-1572 AVRFRLLQNFKQLITLQLNDEADYATIVGKTDGLFPSKFKGRGLIKRD-AIYEFQ-----
TsfC/1-1544 SVRYKVTANFKLSIALTCVDKGEYSNVTGRV-RMEPENKKGRALINVD-NLYEFQ-----
TsgC/1-1293 FVRYKFSVNFKLAVTFQMTDKGDYAGVVGRTEGLEPMKVPGRGLVRAK-PPLEFQ-----
TshC/1-1330 TFPYRIAQNIKQSIVFQMVDSSDYSSIVGRTEGLEPAKVVGRGLVKDQ-MPLEFQ-----
TsiC/1-1522 SVKFKITQSIKLMVALQLNDKYDYVSIVGQTGGLEPEQVKGRGLFRIE-TPLEFQ-----
TsjC/1-1525 SVHMKIKENVAFNIAYNLNDTSEYREIFGRNNGIVPDKISGRGVLRNV-NLLEFQ-----
```

```
EccC/1-1391 HTLVARPALGSTPDNVFECDSVVAAVSRLTSA----QAPPVRRLPARFGVEQVRELASRD
EssC/1-1479 ---IGQPFKHDETKS--YNDQINDEVSAMTEFYKGETPNDIPMMPDEIKYEDYRESLNL-
TscC/1-1328 ---AALPAAGNDEAE--RSLALRSLIQGITVRYPDVHAPLIKKLPERIMLPELMPHAESY
TsdC/1-1563 ---GASLC--ADPSG--EVQAVRDFCGSLAAGWGGTCAKQIPVLPRHVVPSMFNTGEGYD
TseC/1-1572 ---VAQI---TAEHV--PYPSIQAACQKLQEAWKGTAAKRVPILPEQVDHEFLAEYAGPK
TsfC/1-1544 ---TALPVRGENEGA--RAANVKELIEELNSAWKGKHAKPIPIVPDILSLEEAVEELEGN
TsgC/1-1293 ---ASLP---EYEGL--TCEQLLDRFAGLETF----QAKPIPIMPSSIDIGQINKG----
TshC/1-1330 ---GATPVEGESDDQ--IAEGIRVLSGQMKDKWKKKQVKTIAIIPDELSIKEFIENVKSH
TsiC/1-1522 ---TALACKSVNEAE--RVQKLRDLFMKMDESWIGNRAKPIPFVPEKLTIDHLLEHEDSK
TsjC/1-1525 ---TALPVEAE-EFN--WSNEVSDKISSINYSYPNIKVKGIPVLTEVLPLYDFIHGNEFL

EccC/1-1391 TR---QGVG-AGGIAWAISELDLAPVYLNFAENSHLMVTGRRECGRTTTL-ATIMSEIGR
EssC/1-1479 -----PDIVANGALPIGLDYEGVTLQKIKLTEPAMISSENPREIAHIAEIMMKEIDILNE
TscC/1-1328 RPE--RGLNAAFKVPVGLFTDDLEPFELDLREGPHFIVASPMEGGKTSFL-LTWMLSLAY
TsdC/1-1563 ----------ARSIPVGYAKEGAFPVEFDGSRNAMIVSSDDGDAL------YGWLEGIRE
TseC/1-1572 G---------SLTIPIGVEKNSLNVHYYPFGKSYLNMILSAGTEH------LSFVHDLSE
TsfC/1-1544 -----EELE-KYSYTIGIDYTEIEYVYGNLIENPLITVVGKSQTGKSNTI-QSIAHTLAV
TsgC/1-1293 ----------AGKLAIGLANNDLQPVYVDLYTTPIIMVAGEAMSGKSTLL-RSWINVLDE
TshC/1-1330 AGG--QGEQ-KYAFPVGLDWDSTLPFAMDVSSTNNLLLSYAPSVNAELLF-ASLLHSMSA
TsiC/1-1522 -----EFID-NNLLPIGYDTDEAEIVAVDLTGLSSYLILGYEMTGKTNLI-KSLMNTIVD
TsjC/1-1525 DYGLEEEFVYNSRIPVGVNIDEMESVFANFLSIDNMLISGESGCGKTNFL-LSFIMTIAE

EccC/1-1391 LYAPGASSAPPPAPGRPSAQVWLVDPRR---QLLTALGSDYVE--RFAYNLDGVVAMMGE
EssC/1-1479 KY-----------------AICIADSS----GEFKA-YRHQVA--NFAEEREDIKAIHQL
TscC/1-1328 HY------------SPDHLHMYTIDSRYGSRGLAELRELPHVQ--GSASKEEELALLIGR
TsdC/1-1563 VL------------LVQKRNYVVLDAT----GIFSGVEDSRVL--G---QNDEIAEWIEG
TseC/1-1572 FM-----------AQQAGVDVTLIDAEQ---SFIQKNNAGF----QYYSTVKEFEELTGD
TsfC/1-1544 NS------------GVNESKIYLIDSENY--GLLKIKDSNLIK--AYGYKTDESRNIMLE
TsgC/1-1293 VQ------------------VYALDSNGM--GLFEIMSLPYVT--DLAEAEESFIDEMKE
TshC/1-1330 FL------------DSERVKINVFDSS----TVLKSSGNINLEHVHYMRTEGEFSLLTHS
TsiC/1-1522 KL----------------DWKVFVVEKGK---NLQKASSRYQVD--GYINTTEDFDSFIGG
TsjC/1-1525 TK------------NRENNKIYIIDSPER--NMIATSKLNCID--GYLENAEKVADSLAF

EccC/1-1391 LAAALAGR-----------EPPPGLSAEELLSRSWWSGPEIFLIVDDIQQLPPGFDSP--
EssC/1-1479 MIEDLKQR---------EMDGPFE--------------KDSLYIINDFKTFIDCTYIP--
TscC/1-1328 VYDQVQQRGSGEGN------------------------PAILLAIDDADILAKQLNDFM-
TsdC/1-1563 M---LSGRL-----------------------------HADVIVIPSVVQLMVGLTGSV-
TseC/1-1572 LFDLVVYRNNSFKE---ALEEGRE-------VEHF---KQKVIIINSVVALKNALSQLG-
TsfC/1-1544 IKNEIELRKEALNNA--RMTSTGVFNEKEF-LDNM---PLIALFIDDLNDFMLQFSSDVE
TsgC/1-1293 LLDERRSQ---------MAECRRGGGDIKQLMRSW---QQIVFVFDKLSEFTDGDNYAL-
TshC/1-1330 VIEQLQIRKNDSREYVANMDDPDQFNESDYILSRY---PLFIICLPDIKASIERMDSDS-
TsiC/1-1522 LVEEMKLRFKDLKQF--REEDEQVVKEHEY-MKKY---QRIVVLVDDYNEFFEMISDDS-
TsjC/1-1525 IKQEIGERKSILRNL--RMEYGRDANKKI--ISDK---GNIFLIIDVIENFKIRFSDEI-

EccC/1-1391 -LHKAVPFVNRAADV-GLHVIVTRTFGGWSSAGSDPML---RALHQANAPLLVMDADPDE
EssC/1-1479 -EDDVKKLITKGPEL-GLNILFVGIHKELIDAYDKQIDVARKMINQFSIGIRISDQQFFK
TscC/1-1328 IKEQLGTIVRLGRDR-NVHVLLSGVPAD-FPTFGVDWF---NDVKACQSGFLFGTLDPND
TsdC/1-1563 -SDGFKAFLESERYK-GAAVMILASESWRLRGVFDSWF---KAVTANPNGLWIGDGFTSE
TseC/1-1572 -TEKLGLVLEKGDAKYNVTIIFAEQSKNVSSVTFDKWF---KQHIHSGNGIWIGSGITEQ
TsfC/1-1544 IMQIFEGIVQKDKNL-KVALIVAGTEEN-INNYAYSYDFV-KELKSLNYGFFFDN--INN
TsgC/1-1293 -KELLERIVKQERGM-KVAVIAADA----ISDFNGNWDSLGKAIKEEQTGILLGS--IKE
TshC/1-1330 -LQHMERIARFGGGL-GVHLLIGGLAEEIDQLQLVTSLV--PLLAEGESKMLVGGKPFEH
TsiC/1-1522 -SRLVENIVRSGMGL-EVCFVFTADTDTLSPHIGSPLY---NSVFKGMNGILLGGKMDSQ
TsjC/1-1525 -AQELEWIIANCKST-GIHVIVA---DN-MTLFTRAWDGMEKAIKSWETGIIFST--SVD

EccC/1-1391 -GFIRGKMKG------------GPLPRGRGLLMAEDTGVFVQVAATEVRR----------
EssC/1-1479 FRFIQRE---------------PVIKENEAYMVANQAYQKIRWFK---------------
TscC/1-1328 LSFF----RIPFSESGNASGGLKLLPPGQGYYI-KRKFTRVK-AAMPFDAEWSGNRWVTE
TsdC/1-1563 TVFPHTNVAEPFK---------RPMGPHDGVMHGTGRNTLMRLIEPDGKQEQGENGGKGR
TseC/1-1572 FQLKPAKTTSEMR---------EEMPADFAYSLQNGKAVKVKLLNSQKENDDDDE-----
TsfC/1-1544 SRFY--EVNVPYSYKE------KKFENGDGYFIDRGEF---KMIKIPFFK----------
TsgC/1-1293 QGLF--SARLPYGMTE------KEMDNYDGYFIHKSKFVGMRCATIPANDATQSLVLTN-
TshC/1-1330 TTLIGDSYKGDFQELN------RKMQEGEARFVIGTEIKRLKMPTVL-------------
TsiC/1-1522 NIF---DVQMDYKQRS------VALNPGFGYLINRSDYITIKTPQL--------------
TsjC/1-1525 SVFS--SVNIGFENSK------RILGLGEGFLIVNRKAVPVK-LPAPFEGKVKFMNYIDK
```

```
EccC/1-1391 ---------
EssC/1-1479 ---------
TscC/1-1328 IRDRWHVVV
TsdC/1-1563 SVKS-----
TseC/1-1572 ---------
TsfC/1-1544 ---------
TsgC/1-1293 ---------
TshC/1-1330 ---------
TsiC/1-1522 ---------
TsjC/1-1525 LNEKLN---
```

Figure S2. Sequence alignment of the ten representative TsxC sequences, one from each of T7SSa – T7SSj. The region boxed in green is the FHA domains, in purple is the two transmembrane domains and in yellow the four NTPase domains. The consensus sequence for phosphothreonine recognition is given in red under the FHA domain region. The sequences used to generate this figure are WP_003408799.1 (EccC), WP_000549278.1 (EssC), WP_212980188.1 (TscC), WP_052118160.1 (TsdC), WP_213117477.1 (TseC), WP_010963368.1 (TsfC), WP_251417664.1 (TsgC), WP_172455381.1 (TshC), WP_212693241.1 (TsiC) and WP_257675217.1 (TsjC).

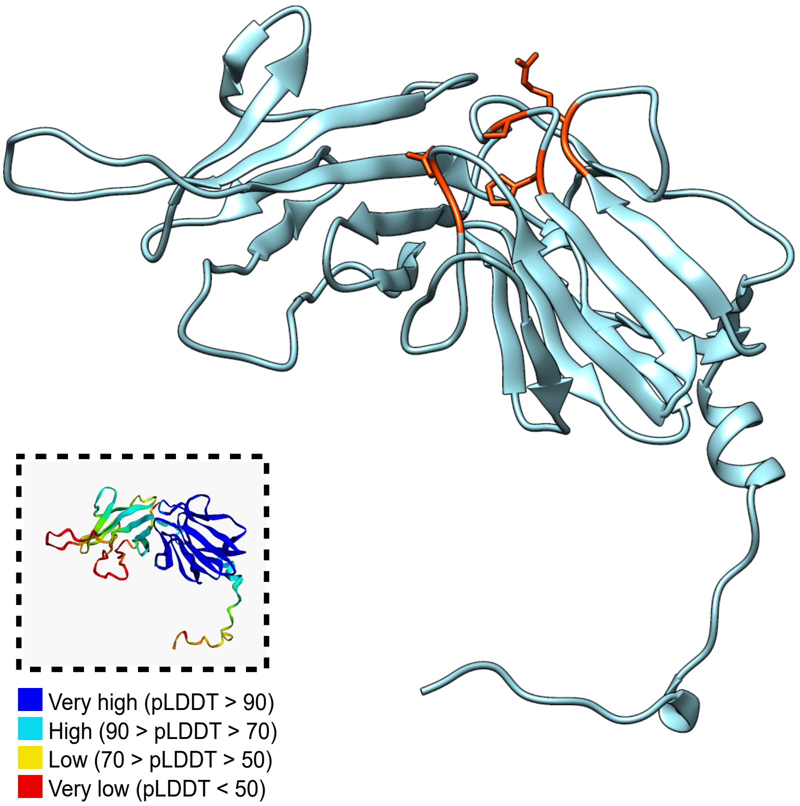

Figure S3. AlphaFold2 model of residues 1-250 of TsdC (WP_052118160.1), showing predicted FHA domains (cyan). Highlighted in red are conserved phosphopeptide-binding residues of the second FHA domain. Model coloured using UCSF Chimera. Inset: model coloured by pLDDT score (per-residue model confidence). Average pLDDT = 77.7.

```
TseJ/258-420 MEDAKFCSACGTAL-------------GGQAGRLEAAAETKEPFAAGPSETEHFSGTTVL
TsfJ/300-468 FMIIAIAVVVAVDVALVIFILL----RGNKVETVADKRRANTSFIKRDKEA----KESKP
TsjJ/332-508 LKVLTISILLGLDLITSVLIVLNFMKKKNKVTFNNVYSRNTESIGKTSNTNSNTTNNIKM

TseJ/258-420 GVEDVGGTTVLEADVYEEPRFPYLVREK--TQEEISVDKPAFRIGKERSYCDYFVSNNNA
TsfJ/300-468 SKREIVTEMSYETELLDS-KTAFLMSKKAGTVERIFINKDSFKIGRISGQADY-ISDNKA
TsjJ/332-508 SKREIVSEMSYSTQLINE-KFPYLLENKKGVVEKIFINKDSFKVGRLSGSVDY-VSDNRA
                                                            GR

TseJ/258-420 ISRSHADIITRGGRYYIIDNNSTNKTYVDGRVIPVMKEIEIFSGTKLRLANEDFTFYI-
TsfJ/300-468 VGKLHAEIRKQNEKYYLIDLTSRNGTFVNGQKINSDELYEIRNGDTIMFANSEFTFAIE
TsjJ/332-508 IGKLHAEIRKVNSDYYVMDLESKNGTFINDKRLESNKLYKMKENDIIKFANSYYTFKFN
              SXXH                NG
```

Figure S4. Amino acid alignment of predicted FHA domains of TsxJ components TseJ (WP_213117486.1), TsfJ (WP_010963365.1) and TsjJ (WP_257675219.1). Phosphopeptide-binding motifs are highlighted in red, with the phosphothreonine binding consensus sequence shown underneath.
