# Supplementary material for: Multiple variants of the type VII secretion system in Gram-positive bacteria
Source: Microlife. 2024 Jun 5;5:uqae013. doi: 10.1093/femsml/uqae013 (PMC11217815; doi:10.1093/femsml/uqae013)
Supplement: uqae013_Supplemental_Files [file uqae013_supplemental_files.zip › Supplementary Data 4.pdf]

Supplementary Data 4

AlphaFold2 models for each of the predicted T7SS components

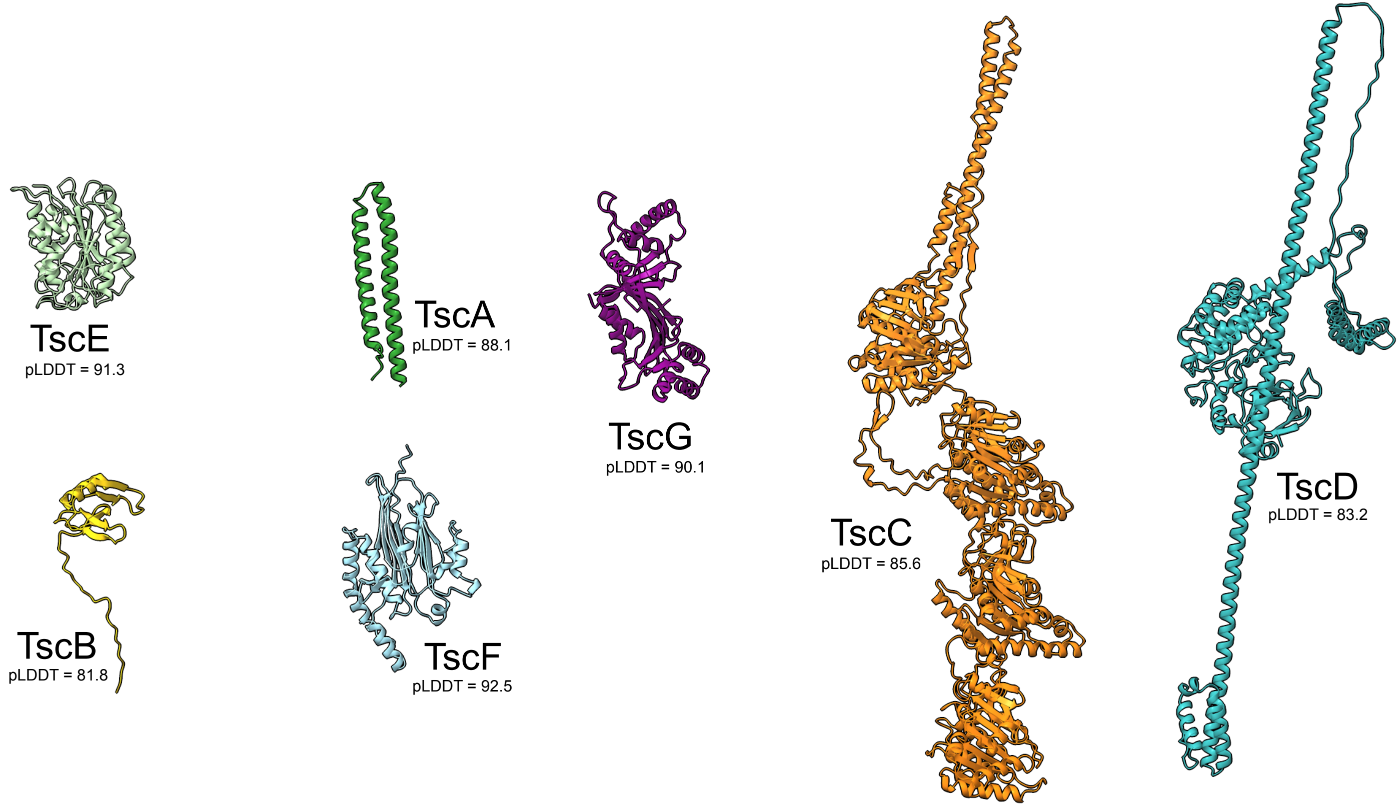
TscE
pLDDT = 91.3
TscA
pLDDT = 88.1
TscG
pLDDT = 90.1
TscC
pLDDT = 85.6
TscD
pLDDT = 83.2
TscB
pLDDT = 81.8
TscF
pLDDT = 92.5

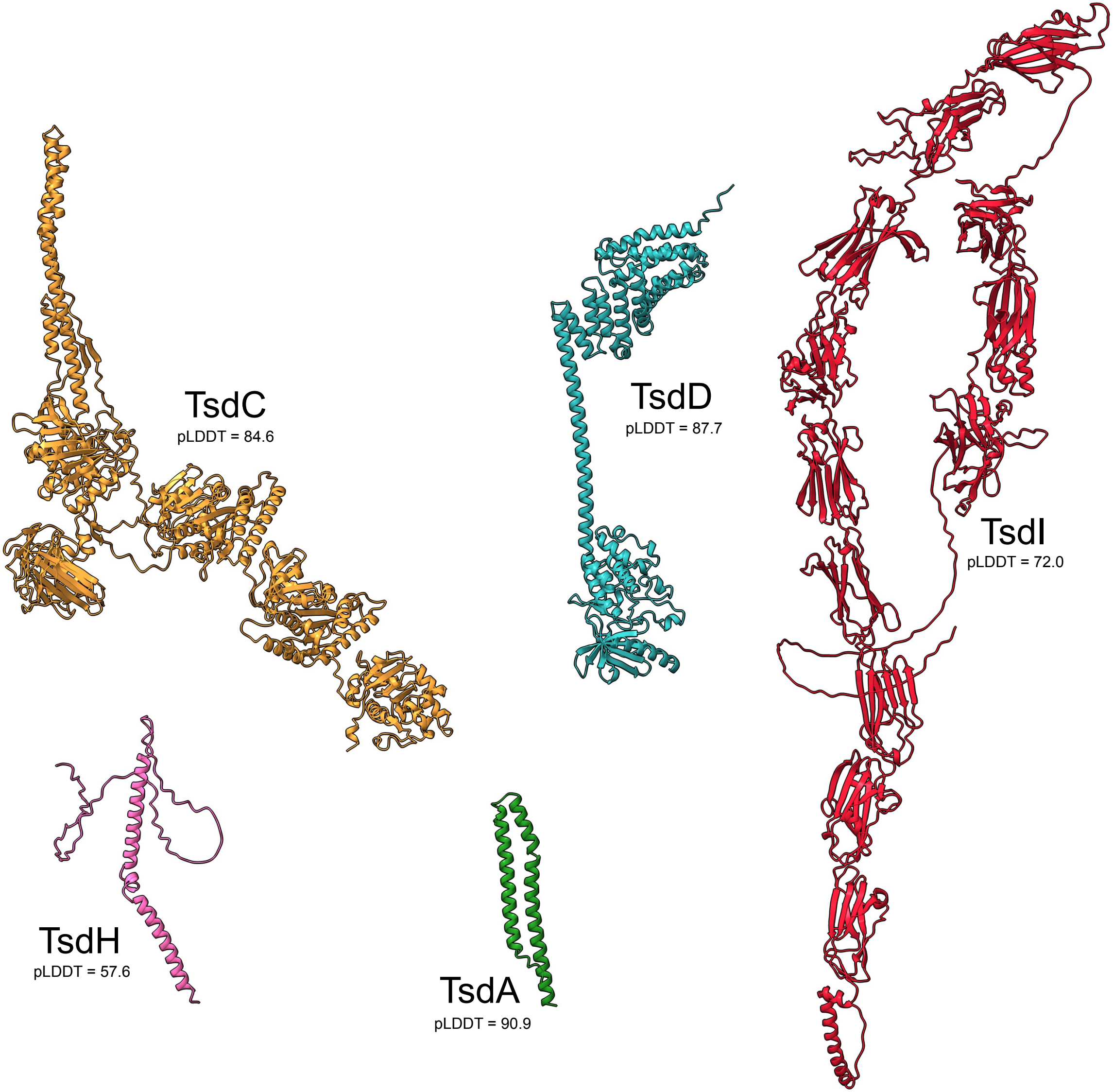

TsdC
pLDDT = 84.6
TsdD
pLDDT = 87.7
TsdI
pLDDT = 72.0
TsdH
pLDDT = 57.6
TsdA
pLDDT = 90.9

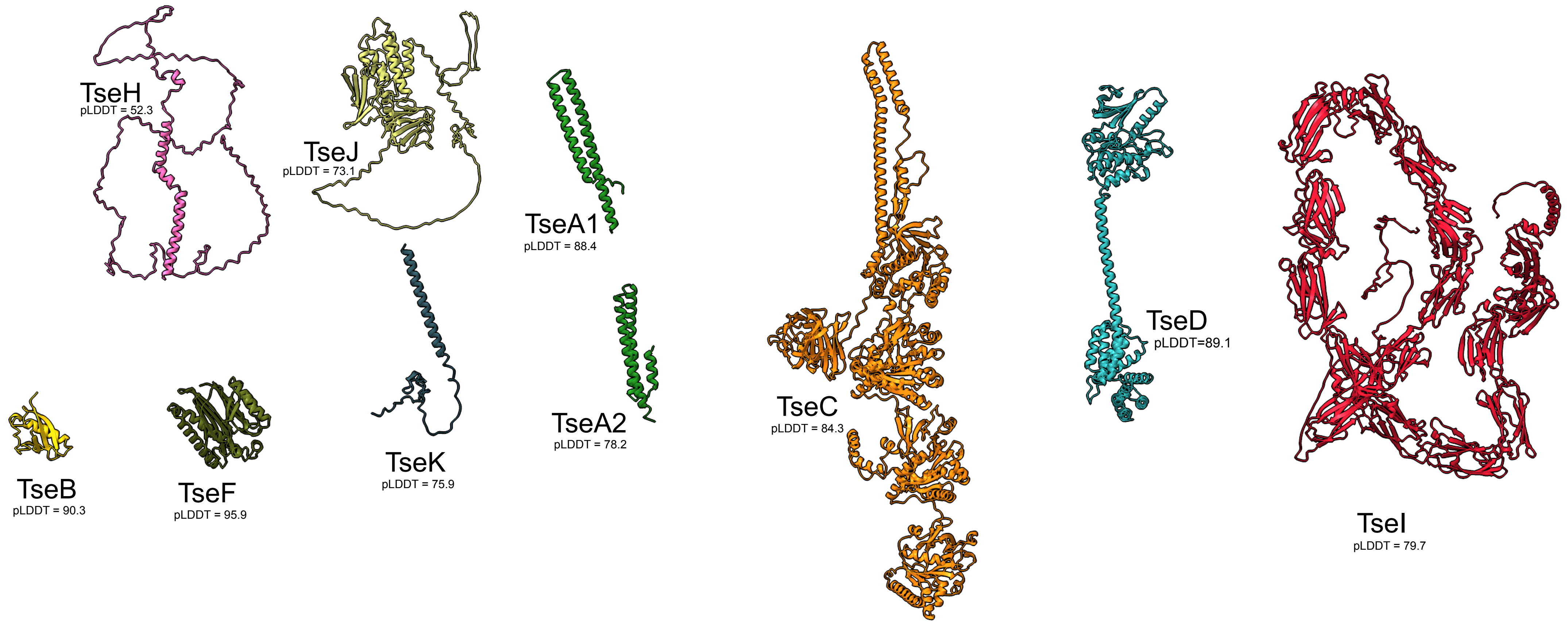
TseH
pLDDT = 52.3
TseJ
pLDDT = 73.1
TseA1
pLDDT = 88.4
TseK
pLDDT = 75.9
TseA2
pLDDT = 78.2
TseB
pLDDT = 90.3
TseF
pLDDT = 95.9
TseC
pLDDT = 84.3
TseD
pLDDT=89.1
TseI
pLDDT = 79.7

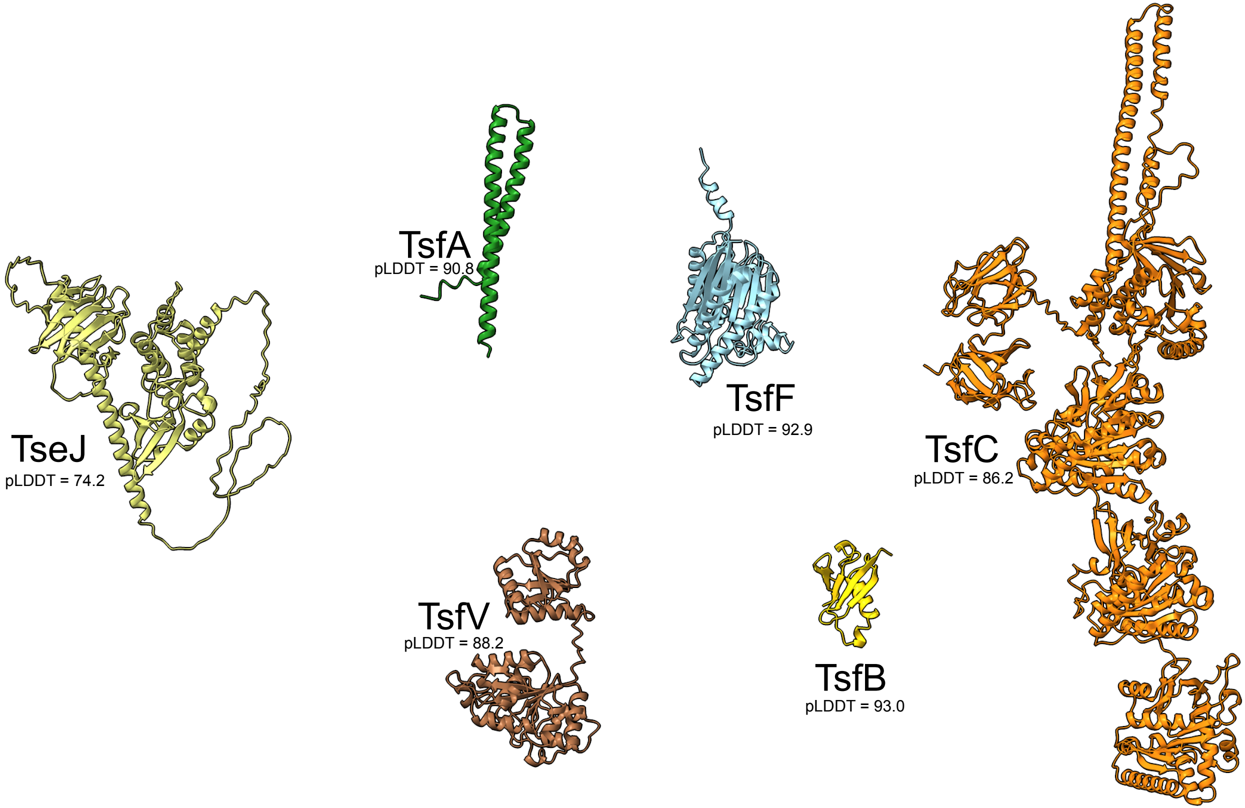
TseJ
pLDDT = 74.2
TsfA
pLDDT = 90.8
TsfF
pLDDT = 92.9
TsfC
pLDDT = 86.2
TsfV
pLDDT = 88.2
TsfB
pLDDT = 93.0

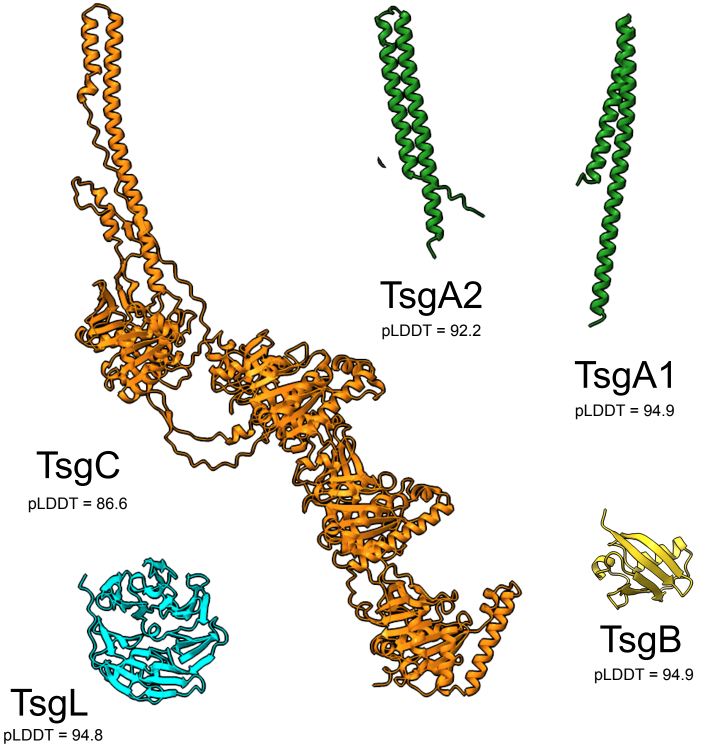
TsgA2
pLDDT = 92.2
TsgA1
pLDDT = 94.9
TsgC
pLDDT = 86.6
TsgB
pLDDT = 94.9
TsgL
pLDDT = 94.8

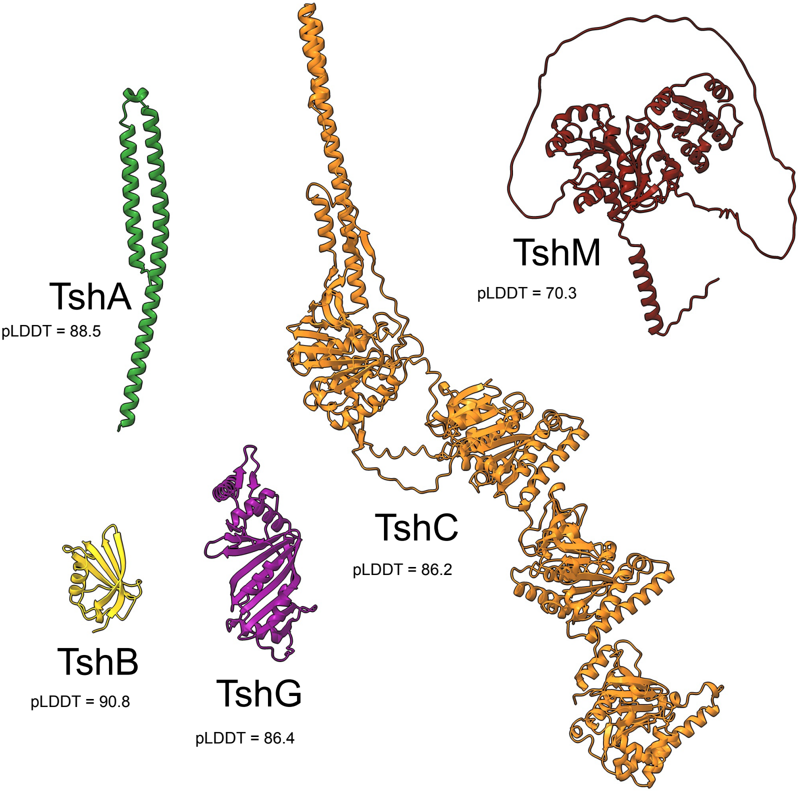
TshA
pLDDT = 88.5
TshC
pLDDT = 86.2
TshM
pLDDT = 70.3
TshB
pLDDT = 90.8
TshG
pLDDT = 86.4

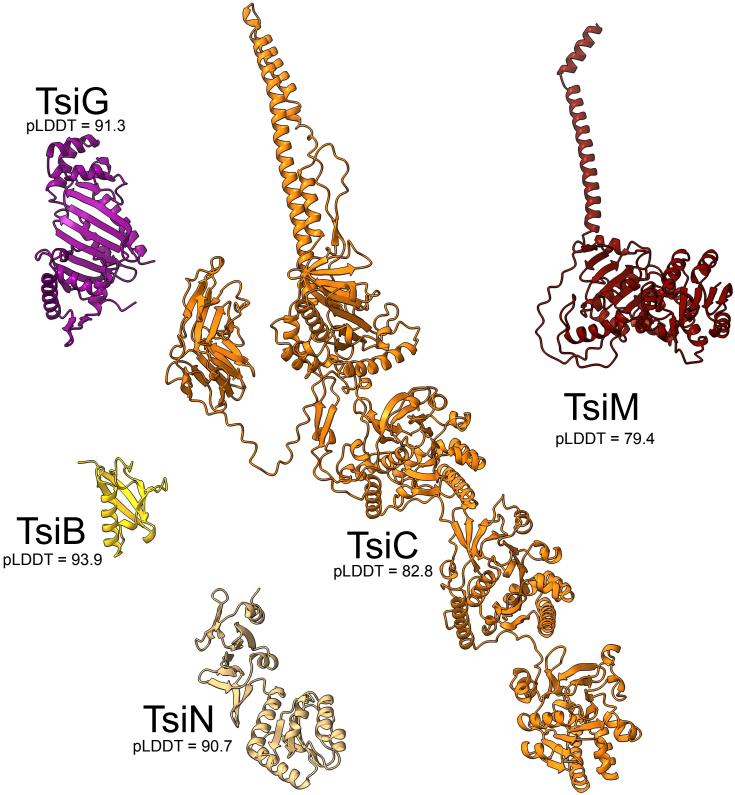
TsiG
pLDDT = 91.3
TsiM
pLDDT = 79.4
TsiB
pLDDT = 93.9
TsiC
pLDDT = 82.8
TsiN
pLDDT = 90.7

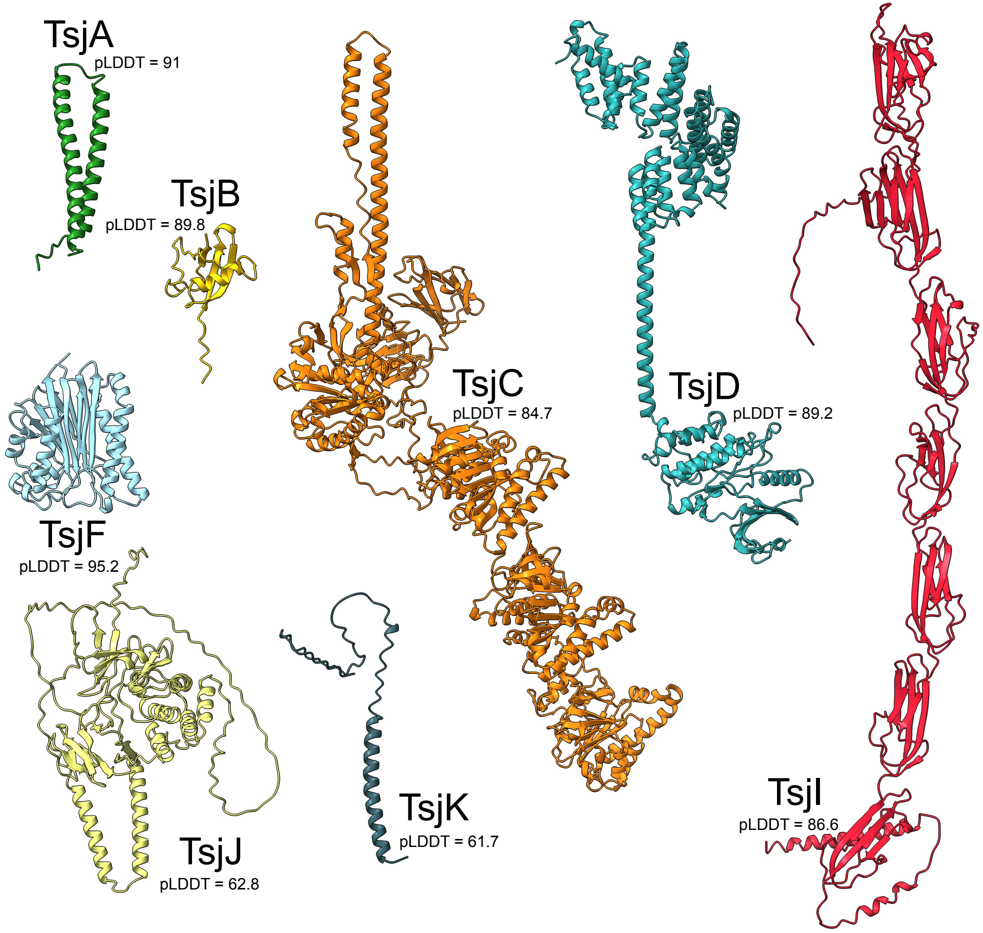
TsjA
pLDDT = 91
TsjB
pLDDT = 89.8
TsjC
pLDDT = 84.7
TsjD
pLDDT = 89.2
TsjF
pLDDT = 95.2
TsjI
pLDDT = 86.6
TsjJ
pLDDT = 62.8
TsjK
pLDDT = 61.7
