# Supplementary material for: Multiple variants of the type VII secretion system in Gram-positive bacteria
Source: Microlife. 2024 Jun 5;5:uqae013. doi: 10.1093/femsml/uqae013 (PMC11217815; doi:10.1093/femsml/uqae013)
Supplement: uqae013_Supplemental_Files [file uqae013_supplemental_files.zip › Table S1.pdf]

	Motif 1 (GR)	Motif 2 (SxxH)	Motif 3 (NG)	Kinase component
TsbC (EssC)	0	0	21	NO
TsdC	100	98	90	YES
TseC	98	99	90	YES
TsfC	0	0	88	NO
TsiC	20	30	90	NO
TsjC	100	100	100	YES

Table S1. Percentage conservation of the three phosphothreonine binding motifs across n representative TsxC FHA domains. nTsbC = 100, nTsdC = 41, nTseC = 152, nTsfC = 16, nTsiC = 10, nTsjC = 8.
