# Supplementary material for: Multiple variants of the type VII secretion system in Gram-positive bacteria
Source: Microlife. 2024 Jun 5;5:uqae013. doi: 10.1093/femsml/uqae013 (PMC11217815; doi:10.1093/femsml/uqae013)

Supplementary Data 1

FlaGs flanking gene analysis on an EssC homologue found in *B. brevis* was used to delineate novel variants of the T7SS, which have been labelled as T7SSc-T7SSj. Annotations for the genes identified in this neighbourhood analysis can be found in Supplementary Data 2.

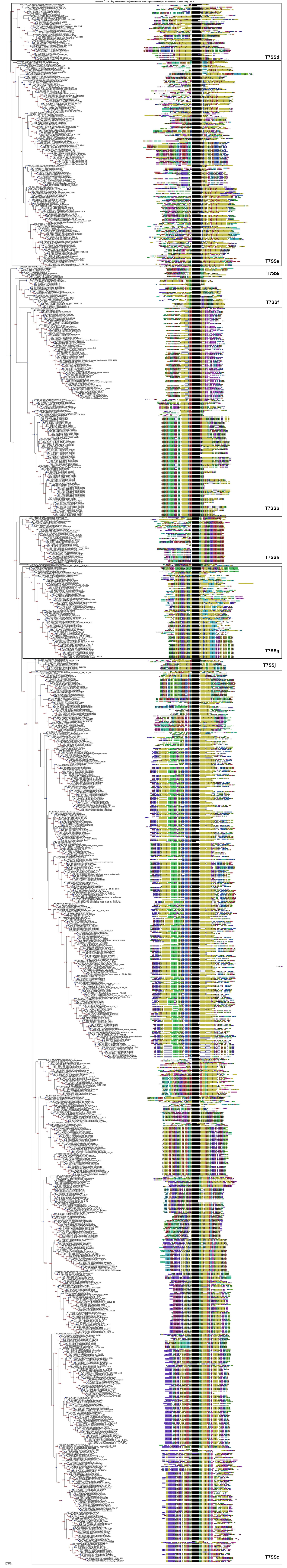

Supplement: uqae013_Supplemental_Files [file Supplementary Data 1.pdf › Supplementary Data 1.pdf]
